# Simultaneous detection of multiple targets for ultrastructural immunocytochemistry

**DOI:** 10.1007/s00418-013-1178-6

**Published:** 2014-01-22

**Authors:** V. V. Philimonenko, A. A. Philimonenko, I. Šloufová, M. Hrubý, F. Novotný, Z. Halbhuber, M. Krivjanská, J. Nebesářová, M. Šlouf, P. Hozák

**Affiliations:** 1 Institute of Molecular Genetics, Academy of Sciences of the Czech Republic, Vídeňská 1083, 14200 Prague 4, Czech Republic; 2 Institute of Macromolecular Chemistry, Academy of Sciences of the Czech Republic, Heyrovského nám. 2, 16206 Prague 6, Czech Republic; 3 Central European Biosystems, s.r.o., Nad Safinou II 365, 25242 Vestec, Czech Republic; 4 Institute of Parasitology, Biology Centre, Academy of Sciences of the Czech Republic, Branišovská 31, 37005 Ceske Budejovice, Czech Republic; 5 Faculty of Nuclear Sciences and Physical Engineering, Czech Technical University in Prague, Břehová 7, 11519 Prague 1, Czech Republic; 6 Department of Physical and Macromolecular Chemistry, Faculty of Science, Charles University in Prague, Hlavova 2030, 12840 Prague 2, Czech Republic

**Keywords:** Immunolabeling, Metal nanoparticles, Electron microscopy, Cell nucleus, Ultrastructure, Phosphatidylinositol-4,5-Bisphosphate (PIP_2_)

## Abstract

Simultaneous detection of biological molecules by means of indirect immunolabeling provides valuable information about their localization in cellular compartments and their possible interactions in macromolecular complexes. While fluorescent microscopy allows for simultaneous detection of multiple antigens, the sensitive electron microscopy immunodetection is limited to only two antigens. In order to overcome this limitation, we prepared a set of novel, shape-coded metal nanoparticles readily discernible in transmission electron microscopy which can be conjugated to antibodies or other bioreactive molecules. With the use of novel nanoparticles, various combinations with commercial gold nanoparticles can be made to obtain a set for simultaneous labeling. For the first time in ultrastructural histochemistry, up to five molecular targets can be identified simultaneously. We demonstrate the usefulness of the method by mapping of the localization of nuclear lipid phosphatidylinositol-4,5-bisphosphate together with four other molecules crucial for genome function, which proves its suitability for a wide range of biomedical applications.

## Introduction

Immunolabeling of biological molecules in situ is indispensable in life sciences and medicine, including diagnostics and pharmacology. Simultaneous detection of several antigens provides valuable information about their localization in cellular compartments and their possible interactions in macromolecular complexes. Fluorescent microscopy enables multiple labeling, but its resolution is often insufficient for an unequivocal localization of the labeled molecules and their assignment to specific cellular compartments. Owing to higher resolution, electron microscopy largely removes such ambiguity. Since pioneering works by Roth and co-workers, it employs gold nanoparticles tagged with immunoglobulin or other bioactive molecules for the detection of molecular targets (antigens) (Roth et al. 1996). However, the number of simultaneously detected antigens is limited to two or three at most. The main limitation is that the gold nanoparticles can only be distinguished by their size which may be varied in a narrow range for the immunodetection to work well.

To increase the number of mutually discernible nanoparticles types within an acceptable size range, two approaches may be employed: discrimination by elemental composition or by shape of the nanoparticle. Several reports have been presented on using nanoparticles of elemental composition different from gold. They can be distinguished from conventional gold nanoparticles by dark-field STEM (Loukanov et al. 2010), energy-dispersive X-ray (EDX) microanalysis (Loukanov et al. 2010), BSE imaging in high-resolution SEM (Vancova et al. 2011), or by electron energy filtering microscopy (Kandela et al. 2007). The results clearly demonstrated the feasibility of such approaches, the drawback being the need for highly specialized and costly equipment not available in most laboratories. To the best of our knowledge, only one group has so far explored the option of distinguishing among nanoparticles by their shape (Meyer et al. 2005, 2010). A clear advantage of this approach is that samples can be routinely analyzed by conventional transmission electron microscopy (TEM) readily available in most laboratories. However, the size of their nanoparticles was often out of the optimal range, and variability of the shapes could also pose a problem.

The present paper describes a procedure of a simultaneous and reliable detection of five different antigens in a cell, based on the use of two ‘conventional’ and three novel nanoparticles that can be easily distinguished in conventional TEM by their size and shape, respectively. This includes their synthesis, conjugation with antibodies, and a labeling efficiency test as a proof of concept.

## Materials and methods

### Synthesis of nanoparticles

Cubic palladium nanoparticles (PdC) were prepared according to procedures of Lim et al. 2009 and Slouf et al. 2012 with modifications. Briefly, an aqueous solution (total volume 11 ml) containing Na_2_[PdCl_4_] (56 mg, 0.19 mmol), l-ascorbic acid (60 mg, 0.34 mmol), polyvinylpyrrolidone (PVP; *M*_w_ = 40 kDa, 106 mg), and KBr (301 mg, 2.53 mmol) was heated to 100 °C for 3 h. The resulting dispersion was diluted with distilled water (1:3 v/v), filtered twice through a microfilter (0.2 μm pore size, Millipore, Cork, Ireland), and purified by dialysis (membrane’s molecular weight cutoff, 3,500 g/mol) against distilled water in order to remove low molecular weight impurities. The procedure results in a colloid which is translucent and chocolate brown in color.

Gold–silver core–shell nanoparticles (AgAu) were prepared by a controlled two-step reduction of soluble salts of Ag^I^ and Au^III^. The principle of the synthesis has been described by Srnová-Šloufová et al. (2000). The AgAu nanoparticles employed in the present study were prepared as follows: First, Ag nanoparticles (the core of AgAu) were prepared by a controlled reduction of AgNO_3_ with NaBH_4_ as described elsewhere (Vlckova et al. 1993). In the second step, 12.5 ml of colloidal solution containing the Ag nanoparticles obtained in the previous step was mixed with 30 ml of double-distilled water in 250-ml Erlenmeyer flask on a magnetic stirrer. Under constant stirring, two solutions were being added, simultaneously and dropwise: (1) 15 ml of HAuCl_4_ solution (4.65 × 10^-4^ M) prepared in double-distilled water, and (2) 15 ml of (NH_2_OH)_2_·H_2_SO_4_ solution prepared in double-distilled water. After 45 min of stirring, a wine-red colored colloid containing gold-coated silver particles (average size under 14 nm) was obtained. In TEM, each particle appears donut-shaped as a bright disk surrounded by a dark annulus as Au is heavier (more electron dense) than Ag.

Small gold nanorods (AuNR) with mean dimensions of 16.3 ± 2.9 × 6.2 ± 0.8 nm were prepared by modifying the usual seeded-growth method in the presence of silver nitrate (Nikoobakht and El-Sayed  2003). The process includes a preparation of monocrystalline gold seeds (2–4 nm) by a fast reduction of gold(III) salt in the presence of hexadecyltrimethylammonium bromide (CTAB), and adding them into the growth solution of gold(I) complexed to CTAB in the presence of silver(I) in aqueous solution. First, a solution of small gold seed nanoparticles was prepared by a fast reduction of gold salt (HAuCl_4_) by sodium borohydride (NaBH_4_) as follows: 4.7 ml of a water solution of CTAB (0.1 M) and HAuCl_4_ (0.25 mM) (concentrations calculated for a total volume of 5 ml) was stirred for 5 min at 30 °C to allow for a complete dissolution of CTAB and its complexation with the gold salt. A 0.3 ml volume of freshly prepared, ice-cold, 10 mM NaBH_4_ was then added under vigorous stirring and stirred for another 2 mins. The seed solution was left overnight at 40 °C to allow for a complete decomposition of NaBH_4_. Growth solution was prepared by a subsequent addition of 50 μl of 0.1 M HAuCl_4_, 120 μl of 0.02 M silver nitrate (AgNO_3_), and 100 μl or 0.1 M ascorbic acid to 16.65 ml of 0.22 M aqueous solution of CTAB. Finally, the small gold nanorods were prepared by adding 2 ml of the seed solution into the growth solution and kept at 25 °C, this being the temperature triggering the growth of AuNRs, which is complete in ca 2 h. The procedure resulted in AuNRs with dimensions stated above, and 18.2 MΩ Milli-Q water was used during the synthesis. The glass sample vial (30 ml) used for the synthesis was treated with Sigma Cote to inhibit adsorption of reactants to the glass walls during the synthesis. All reagents were obtained from Sigma-Aldrich.

### Conjugation of antibodies to nanoparticles

The pH value of the PdC and AgAu colloid dispersion was adjusted to 8.5 with 0.1 M NaOH, and 90 μg of the antibody was added to 1 ml of the colloid dispersion which was then vortexed at 1,400 rpm for 1–2 min. To prevent aggregation and nonspecific binding, bovine serum albumin (BSA) was added (final concentration 0.25 %), and the solution was again vortexed at 1,400 rpm for additional 5 min. The mixture was then incubated at 4 °C for 1 h, in some cases overnight. In order to remove any unbound antibody, the nanoparticles were then centrifuged (PdC 6,600 g; AgAu 4,700*g*) in 1-ml aliquots for 1 h at 4 °C in 10–30–40 % glycerol gradient prepared with PBTB (phosphate-buffered saline with 0.1 % Tween 20 and 1 % w/v BSA). The 30 % glycerol fraction was extracted and stored at 4 °C as a stock solution. For subsequent immunolabeling, it was diluted 1:7 (v/v) in PBTB.

AuNR colloid was first diluted 1:1 (v/v) with double-distilled water in order to reduce CTAB level and centrifuged at 120,000*g* for 30 min at 30 °C. Fifteen microliters of the pellet was diluted in 700 μl of double-distilled water. The colloid was then mixed 1:1 (v/v) with an appropriate antibody solution in double-distilled water (final antibody concentration 60 μg/ml), shaken for 1 min, and after adding BSA (final concentration 0.25 % w/v) shaken for further 5 min. The conjugate was then spun down for 90 min at 120,000*g*. About 20 μl of the pellet was used for immunolabeling.

### Cell culture and LR White resin embedding

HeLa cells were cultured in a suspension in 150 cm^2^ tissue culture flasks (Techno Plastic Products AG, Trasadingen, Switzerland) in Eagle’s minimum essential medium (S-MEM, 0.15 % NaHCO_3_, Sigma-Aldrich, St. Louis, MO, USA) supplemented with 5 % fetal bovine serum (Gibco, Grand Island, NY, USA) at 37 °C in a humidified 5 % CO_2_ atmosphere.

Cells were fixed for 40 min at room temperature in 3 % paraformaldehyde and 0.1 % glutaraldehyde in Sörensen buffer (SB; 0.1 M Na/K phosphate buffer, pH 7.3). Upon washing in SB (including 20 min of incubation in 20 mM glycine in SB), the cells were centrifuged into 1 % low-melting agarose type VII (Sigma) in SB. The agarose-embedded cell samples were quickly dehydrated in a pre-cooled ethanol series (30, 50, 70, 96, and 100 %) and embedded in LR White resin (Polysciences Inc., Warrington, PA, USA) with polymerization under UV light for 48 h at 4 °C.

### Immunolabeling and electron microscopy

Ultrathin 80-nm sections were immunolabeled in a standard way (Hozak et al. 1994) with some minor modifications. Briefly, the sections were placed on poly-l-lysine-coated 200 mesh-gilded grids and immersed in 20–25 μl drops of incubation solutions. The sections were first blocked for 10 min in 1 % normal goat serum in PBTB (i.e., PBS containing 0.1 % Tween 20 of EM grade and 1 % BSA), then incubated for 1 h with a mixture of primary antibodies diluted in PBTB to a concentration of 5–10 μg/ml, washed for 3 × 5 min in PBT (i.e., PBS containing 0.005 % Tween 20 of EM grade), incubated for 45 min with a mixture of secondary antibodies diluted in PBTB, and washed again for 2 × 5 min in PBT, and for 2 × 5 min in double-distilled water. After air-drying, the samples were contrasted for 4 min with a saturated solution of uranyl acetate in water. The entire procedure was performed at room temperature. For controls, sections were incubated in the same way with omitting the primary antibody, and cross-reactivity between antibodies was also controlled with incubating the sections only with one primary antibodies followed by the three ‘incorrect’ secondary antibodies.

The following primary antibodies were used: anti-PIP_2_ mouse monoclonal IgM (Z-A045, clone 2C11 from Echelon Biosciences Inc.), anti-Sm (Smith antigen), purified human autoimmune antibody (RayBiotech), anti-B23 (nucleophosmin) rabbit polyclonal antibody (ab15440 from Abcam), anti-*γ*-actin mouse monoclonal IgG (#A8481 from Sigma), and guinea pig anti-SMC2 kindly provided by W. Earnshaw (Saitoh et al. 1994).

The following secondary antibodies conjugated to nanoparticles were used: goat anti-mouse IgG (Fcγ-specific) coupled to 6-nm gold particles and donkey anti-guinea pig IgG coupled to 12-nm gold particles (purchased as ready-to-use conjugates from Jackson Immunoresearch), goat anti-human IgG (Invitrogen) conjugated to the cubic-shaped PdC nanoparticles, goat anti-rabbit IgG (Invitrogen) conjugated to the core–shell AgAu nanoparticles (imaged as donuts), and goat anti-mouse IgM (Invitrogen) conjugated to the rod-shaped AuNR nanoparticles.

Sections were examined, and images acquired either with Philips Morgagni electron microscope equipped with a CCD Mega View III camera or with Tecnai 20 G2 electron microscope equipped with Gatan electron energy filter (GIF Tridiem) and Gatan CCD camera, either in a non-filtering mode or using contrast enhancement by zero-loss filtering. For quantification of the labeling density, 20 random images of cell sections were taken per each experimental and each control group. The area of the relevant compartments and the number of gold particles were measured on the images using plug-ins to Ellipse program (ViDiTo, Košice, Slovakia).

### Mapping of nuclear compartments

For spatial statistics of immunogold labeling patterns, we used special plug-ins (for details see Philimonenko et al. 2000; Schofer et al. 2004; http://nucleus.img.cas.cz/gold) developed earlier for the Ellipse program (ViDiTo, Košice, Slovakia). The plug-ins made it possible to evaluate clustering and co-localization patterns detected by immunogold labeling, and to identify and map significant labeling of cellular compartments. The nanoparticles were detected as points (dots) in the image, and their density was evaluated using the kernel density estimate method employing a conical kernel function. Thresholding was then used to segment the image, thus delineating the areas with immunogold particles’ density above the background level (Schofer et al. 2004). Identification of the labeled compartments was performed on composite images obtained by a multiple alignment (‘stitching’) of 25–36 high-resolution images per nucleus. This approach combines the advantage of high-resolution images needed to detect the gold particles with the convenience of a zoomed-out image visualizing the entire nucleus. The plug-ins were modified for working with five classes of objects (types of labeling), as the previous versions were designed for maximum of three classes.

## Results

### Synthesis and characterization of metal nanoparticles with distinct shapes

**Fig. 1:**
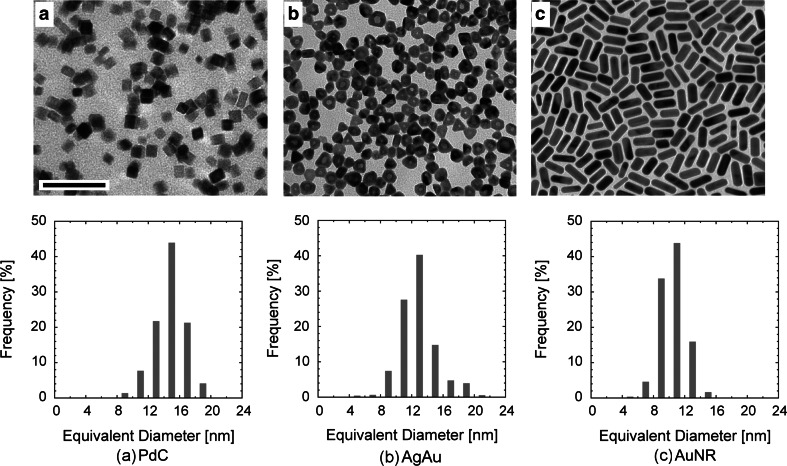
Morphology of novel metal nanoparticles. **a** Cubic palladium nanoparticles (PdC), **b** core–shell donut-like silver-gold nanoparticles (AgAu), **c** rod-like gold nanoparticles (AuNR). The* upper panel* shows TEM micrographs, and the* lower panel* the size-distribution histograms for respective particle types. *Scale bar* 50 nm

The particles for immunodetection should fulfill several criteria: (1) size ideally in the 5–15 nm range; (2) narrow size distribution; (3) stability in solution; (4) good contrast and stability in TEM; (5) chemically defined surface with no contamination of stabilizing agents; (6) the possibility to link biomolecules to the surface of the nanoparticles. None of the commercially available metal nanoparticles, except for gold nanoparticles, fulfill all of the above six criteria. Hence, we significantly modified the existing procedures of nanoparticle synthesis in order to obtain palladium nanoparticles of cubic shape (PdC), gold rod-like nanoparticles (AuNR), and nanoparticles with a silver core and a gold shell (AgAu) with a donut-like appearance in TEM (Fig. 1). All three types of our novel particles were found to be readily discernible among themselves as well as from the commercially available 6- and 12-nm gold nanoparticles. We characterized obtained nanoparticles to check whether they meet all the above criteria for the use in immunodetection. The size distribution for each nanoparticle type was determined from TEM micrographs, as described in Slouf et al. (2012) for palladium nanocubes, and the results are presented in Fig. 1. The mean equivalent diameter of PdC nanoparticles was 14.85 ± 1.91 nm, AuNR nanoparticles 10.49 ± 1.60 nm, and AgAu nanoparticles 13.31 ± 2.38 nm. The microphotographs and distribution histograms in Fig. 1 demonstrate that the distribution of the sizes and shapes for each type of the nanoparticles conforms to the quality requirements. The stability of the nanoparticles in the course of observation in TEM was tested by continuous exposure to electron beam at 200 kV with the dose 50,000 ē/nm^2^ per 1 s for 15 min, and size and density of the nanoparticles after illumination were statistically compared to non-illuminated nanoparticles. The size and contrast of the nanoparticles remained constant upon illumination. The stability of colloidal solutions of the nanoparticles was monitored for up to 6 months for each colloid. All three solutions appeared to be stable: The color of the solution did not change, no precipitation occurred, and the appearance of the nanoparticles in TEM did not change.

For the nanoparticles to be applicable as labels for ultrastructural detection, they need to be coupled to biomolecules targeting them to the molecules of interest. We conjugated our nanoparticles non-covalently to secondary antibodies, allowing us to use a number of primary antibodies to detect the molecule of interest, increasing flexibility. While using a standard protocol as a basis, we modified the conjugation conditions for each type of our nanoparticles, varying the concentrations of the colloid and the antibody, buffer and blocking conditions, and the purification of the resulting conjugates. The colloid solutions of PDC and AgAu nanoparticles did not contain any components interfering with the antibody conjugation and could be used for conjugation directly after the pH adjustment; in the case of AuNR nanoparticles, the concentration of CTAB had to be reduced for successful conjugation, as described in ‘Materials and methods’ section.

**Fig. 2:**
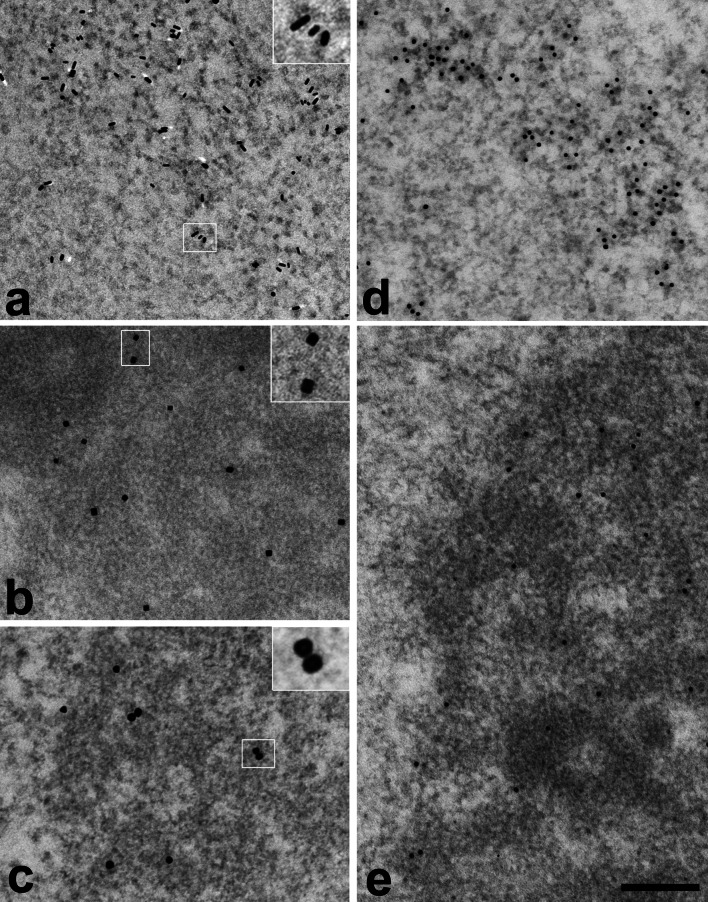
The labeling pattern using novel nanoparticles conjugates corresponds to typical pattern obtained using commercial conjugates. Single immunolabeling on ultrathin sections of LR White-embedded HeLa cells.** a**–**c** Immunolabeling using our novel conjugates, the inserts show magnified view of the nanoparticles from outlined area; **d**, **e** parallel positive control incubations using commercial conjugates with 12-nm gold particles. **a**, **d** Labeling of PIP2; secondary antibodies conjugated with AuNR **a** or 12-nm gold nanoparticles (commercial conjugate; **d**). Characteristic labeling of interchromatin granules clusters is very similar in both cases. **b**, **c**, **e** Labeling of B23; secondary antibodies conjugated with PdC nanoparticles **b**, AgAu nanoparticles **c**, or 12-nm gold nanoparticles (commercial conjugate; **e**). Characteristic labeling of the granular component (GC) of the nucleolus is very similar in all three cases

**Fig. 3:**
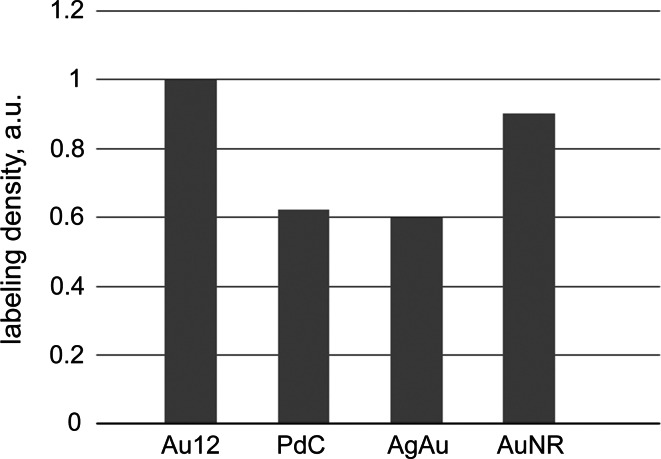
Comparison of labeling density using novel nanoparticles conjugates and commercial conjugates. The histogram represents labeling densities normalized to the labeling density of the same antigen using commercial 12-nm gold conjugate

The applicability of each conjugate to ultrastructural immunodetection was first tested in a single-labeling procedure on ultrathin sections of LR White-embedded HeLa cells, with positive and negative controls. We found that in a standard immunolabeling procedure, the performance of our conjugates was similar to that of commercially available secondary antibodies coupled to gold particles of roughly the same size as our novel nanoparticles (Figs. 2, 3). The microphotographs demonstrate the same labeling pattern in nuclear compartments while using commercial gold conjugates or our nanoparticles conjugates. The labeling density values are also comparable, as shown by histogram in Fig. 3. The background labeling of the samples, incubated in the same way but without the primary antibody, was negligible, ranging between various grids typically between 0.5 and 0.8 particles per μm^2^. Taking into account the usual density labeling, the background staining contributed only about 1–3 % to the overall labeling densities. One can conclude that these nanoparticles have an excellent specific-to-nonspecific labeling ratio.

### Application of nanoparticles conjugated to antibodies in multiple ultrastructural immunolabeling

Having the five reagents at hand, we applied the method in our research. Nuclear compartmentalization is fundamental for correct functioning of the genome. We studied the localization of a nuclear lipid, phosphatidylinositol-4,5-bisphosphate (PIP_2_) in the nuclear compartments by means of multiple immunolabeling using our system of novel nanoparticles. PIP_2_ is a multifunctional lipid that was first described in the plasma membrane. It participates in signal transduction pathways regulating numerous processes in eukaryotic cells, and its presence has also been demonstrated in the cell nucleus. However, much less is known about the details of its nuclear function (Barlow et al. 2010).

**Fig. 4:**
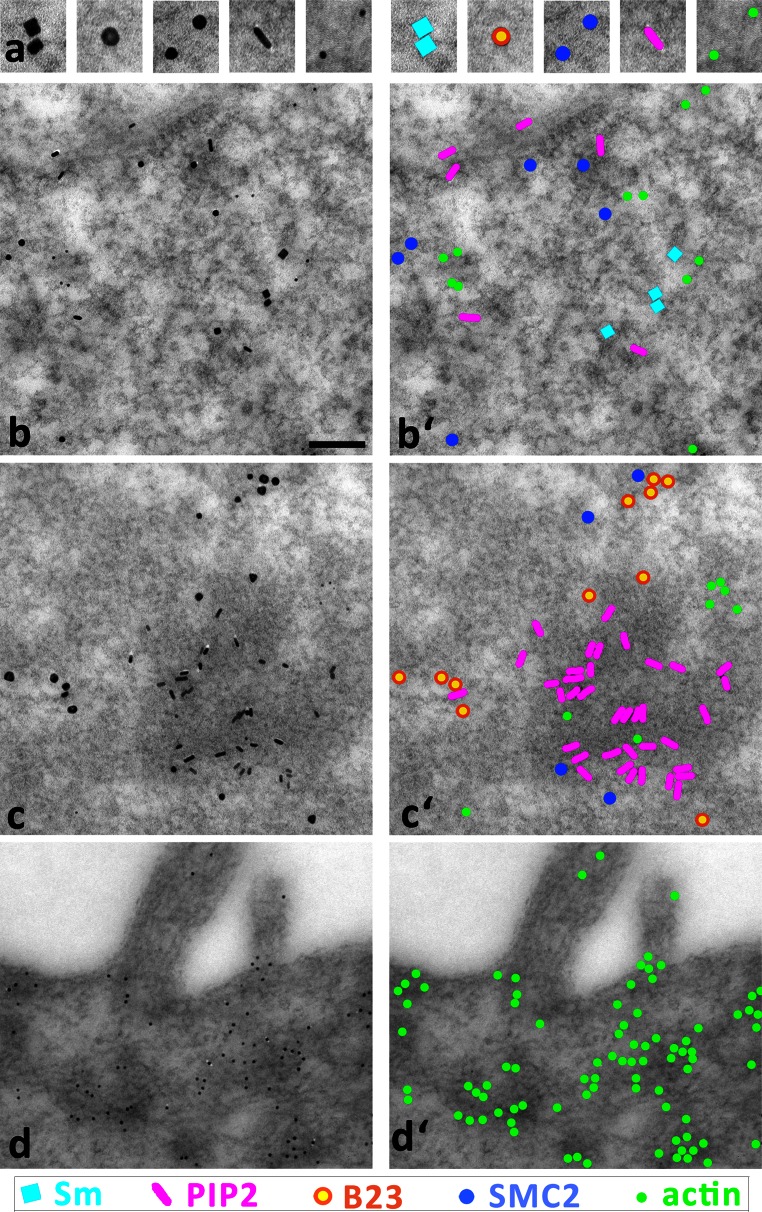
Demonstration of simultaneous ultrastructural detection of five different biomolecules (antigens) on ultrathin sections of HeLa cells. **a** Electron micrographs of individual types of nanoparticles (three of them newly developed). **b**–**d** examples of a fivefold immunolabeling in nucleoplasm **b**, nucleolus **c**, and cytoplasm **d**. Color-coded views are shown in (**a’–d’**). The color legend under the image panel shows the antigens labeled by each particles type. *Scale bar* 100 nm

We established a set of antibodies recognizing five cellular antigens (PIP_2_, B23, actin, Sm protein, and SMC2), in such a way that they could be recognized by different (secondary) antibodies without a cross-reaction (for details, see “Materials and methods”). The results are shown in Fig. 4. The upper panel (a) demonstrates individual particles at high magnification; our novel nanoparticles are easily discernible by their distinct shapes. For clarity, the right half of the image plate shows the same views with particles color-coded. We present typical examples of immunolabeling in nucleoplasm (Fig. 4b), nucleolus (Fig. 4c), and cytoplasm (Fig. 4d). In nucleoplasm, we typically encountered four types of labeling: PIP_2_, Sm, SMC2, and actin. This corresponds to the already known localization of these molecules (Biggiogera et al. 1989; Eliceiri and Ryerse 1984; Yildirim et al. 2013; Sobol et al. 2013; Dingova et al. 2009; Kyselá et al. 2005; Philimonenko et al. 2004). In the nucleolus, we observed abundant PIP_2_ labeling in the dense fibrillar component (DFC) and at the border of fibrillar centers (FC), while B23 was localized predominantly in the granular component (GC). Some SMC2 labeling was present at the peripheral part of the nucleolus, possibly representing incoming chromatin strands, and actin clusters were often observed. The border of cytoplasm typically featured abundant actin labeling in the cortical layer. We can conclude that as in the case of single labeling of individual proteins with our novel nanoparticle conjugates, the distribution of relevant biomolecules is as expected, yet the background nonspecific labeling remains negligible, which documents the specificity of our secondary detection system.

**Fig. 5:**
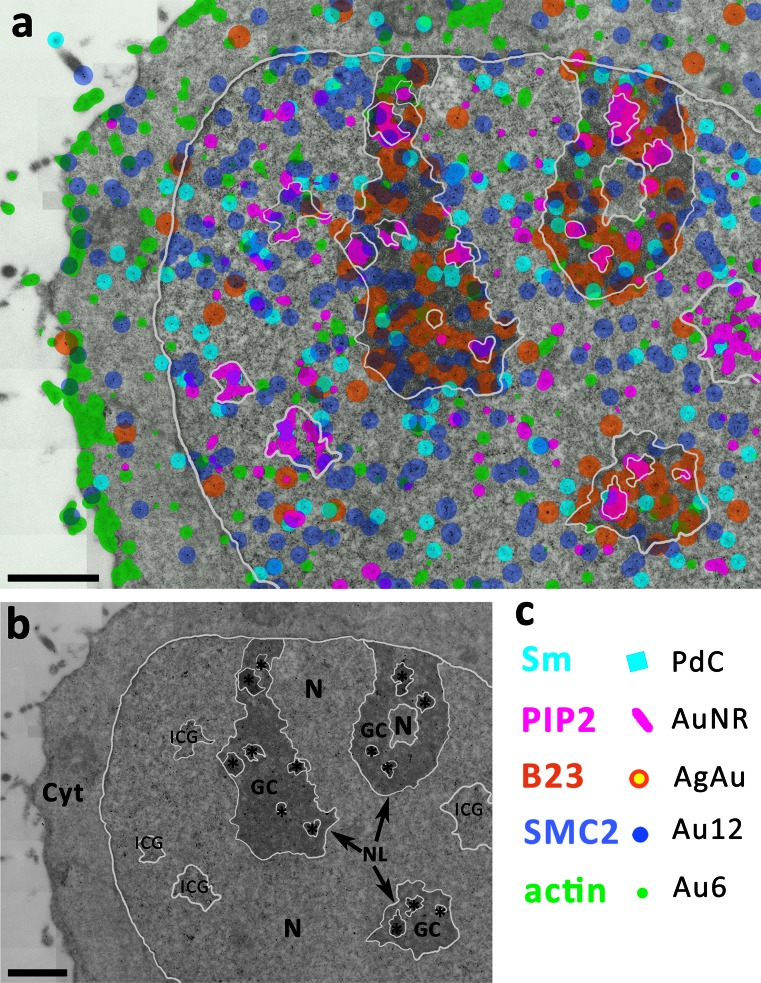
Ultrastructural mapping of all five examined antigens (*Sm, PIP*_2_,* B23, SMC2, actin*) in a HeLa cell (zoom-out view). **a** Statistically significant densities of individual biomolecules (antigens) are highlighted by color-coding in a zoom-out electron micrograph obtained by ‘stitching’ of 70 individual images. Main cellular compartments are delineated with a *white line*. **b** An overview highlighting the delineated compartments.* Cyt* cytoplasm,* ICG* interchromatin granules,* GC* granular component of the nucleolus,* N* nucleoplasm, asterisk–fibrillar center and dense fibrillar component of the nucleolus. **c** Color-coding. *Scale bars* 1 μm

**Fig. 6:**
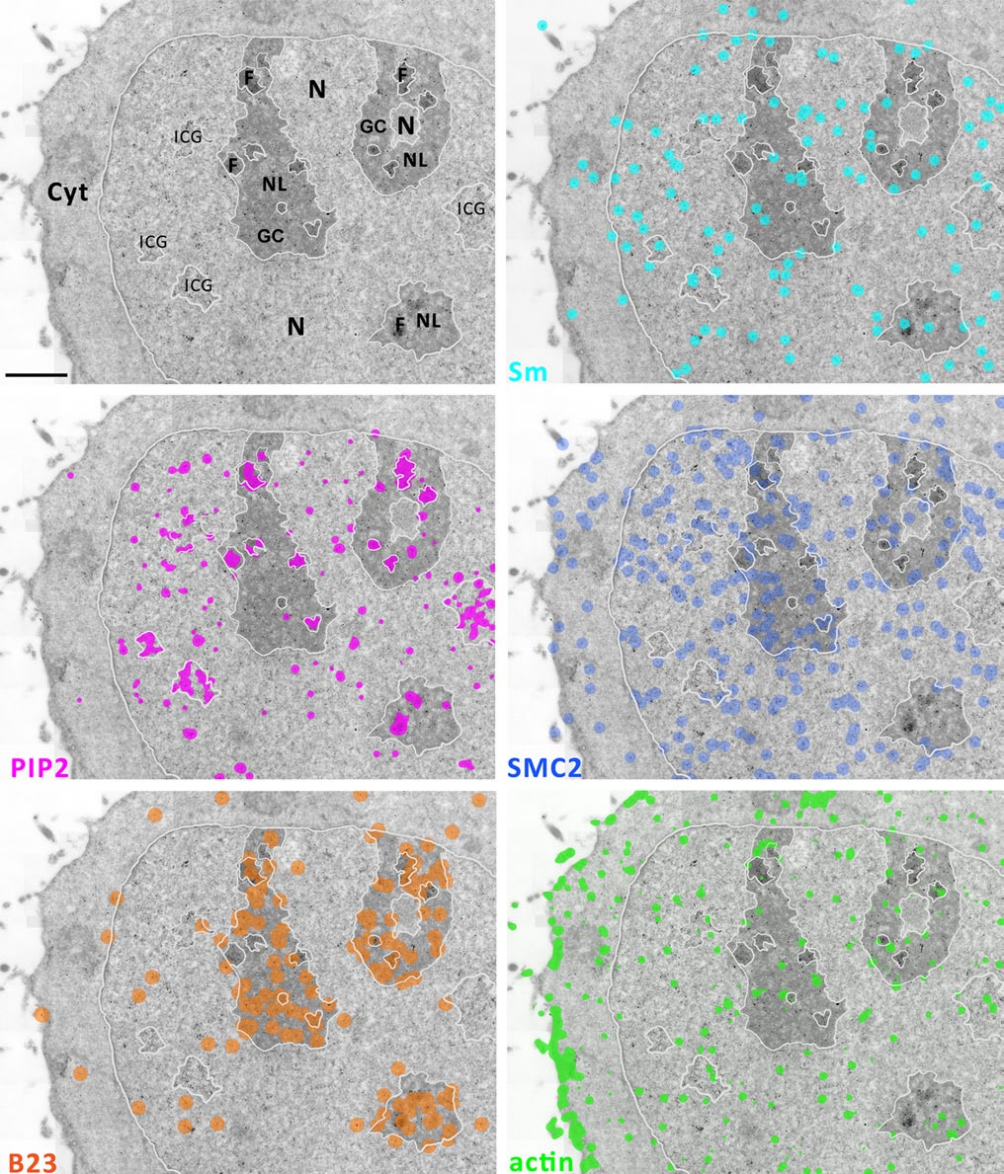
Ultrastructural mapping of single antigens distribution in HeLa cell on a macroscopic scale. Mapping of areas with statistically significant density of respective antigens highlighted by colors on a stitched electron microscopic image. Main cellular compartments are delineated with a *pale*-*gray line*.* Cyt* cytoplasm,* N* nucleoplasm,* ICG* interchromatin granules,* NL* nucleolus,* F* fibrillar center +dense fibrillar component,* GC* granular component. *Scale bar* 1 μm

To map cellular compartments occupied by the antigens of interest, we used a previously developed algorithm. The nanoparticles were detected as points (dots) in the image, and their density was evaluated using the kernel density estimate method. Thresholding was then used to segment the image, thus delineating the areas with immunogold particles’ density above the background level (Schofer et al. 2004). To observe the relative localization of the labeled molecules in an overall view of a larger portion of the cell, we performed multiple-image alignment (also referred to as ‘stitching’) of several adjacent images, yielding a single high-resolution image of a larger area. Figure 5 shows the patterns and mutual localization of five simultaneously labeled antigens on such composite image, with main cellular compartments delineated and nanoparticles color-coded. Distribution of individual antigens, as shown also in Fig. 6 for better orientation, corresponds to the patterns known from the literature (Biggiogera et al. 1989; Eliceiri and Ryerse 1984; Yildirim et al. 2013; Sobol et al. 2013; Dingova et al. 2009; Castano et al. 2010). The areas of dense PIP_2_ labeling (purple) are observed in the FC and DFC in nucleoli and interchromatin granule clusters, along with smaller foci in nucleoplasm and cytoplasm. As expected, B23 (orange) is neighboring the PIP_2_-rich areas in nucleoli, occupying predominantly the GC. Actin (green) is heavily localized in the cortical layer of cytoplasm and also scattered in small foci in the nucleus, nucleolus, and cytoplasm. Sm protein (turquoise) is also found in small clusters throughout the nucleoplasm, presumably involved in splicing. SMC2 being a protein bound mainly to chromatin is localized throughout the nucleus and found also in the nucleoplasm. Co-localizations within small structures at distances <100 nm were observed in some cases. However, the majority of patterns constituted of PIP_2_-positive foci in a close contact with Sm-, actin-, and/or SMC2-rich foci, forming complementary 3D domains.

## Discussion

In order to establish a tool for simultaneous labeling of more than three antigens, we prepared three types of metal nanoparticles differing by their shapes and therefore readily distinguishable by TEM from each other, and from commercially available spherical gold nanoparticles of various sizes usually used for immunodetection. The novel nanoparticles—cubic palladium nanoparticles (PdC), core–shell siver–gold nanoparticles (AgAu), and gold nanorods (AuNR)—were characterized in detail to assess their suitability for immunodetection. The average size of the nanoparticles calculated as equivalent diameter ranged from 10.5 to 14.9 nm, which is within the suitable range for immunolabeling (Roth 1996; Mayhew and Lucocq 2011; Slouf et al. 2011), and the size distribution was narrow. The particles demonstrated good stability upon exposure to electron beam in TEM, as well as in the colloid solution over 6-month period tested. Good contrast and distinct shapes made them very convenient for TEM observation and readily distinguishable from standard spherical gold nanoparticles. All three types of nanoparticles could be successfully non-covalently conjugated to antibodies, demonstrating that the surface of nanoparticles is free of contaminants which would eventually prevent binding of the biomolecules. The obtained secondary antibody conjugates were fully functional, as demonstrated by the results of immunolabeling on ultrathin sections. Somewhat lower labeling density by PdC (14.85 ± 1.91 nm) and AgAu (13.31 ± 2.38 nm) nanoparticles corresponded apparently to their somewhat larger size as compared to 12-nm gold nanoparticles. At the same time, the smaller AuNR particles (10.49 ± 1.60 nm) performed identically to commercial 12-nm gold particles. Taken together, the characterization of these novel nanoparticles presented here demonstrates their high quality and suitability for application in immunodetection.

Using this tool, we performed a proof-of-concept testing of the multiple simultaneous immunolabelling with multiple types of nanoparticles. Five cellular antigens—PIP_2_, B23, actin, Sm protein, and SMC2—were immunolabelled in parallel. Describing all the labeling patters would be out of the scope of this paper, and we will therefore discuss only briefly the observed patterns while concentrating on the method itself. As the role of PIP2 in the cell nucleus is poorly understood, we were especially interested in spatial interactions of PIP2-containing structures with the other four proteins with established cellular functions. Co-localizations of two or three types of labeled molecules within small structures at distances <100 nm were observed in some cases. However, the majority of patterns constituted of PIP_2_-positive foci in a close contact with Sm–, actin-, and/or SMC2-rich foci, forming complementary 3D domains. As the mechanism underlying PIP_2_ compartmentalization in the cell nucleus remains largely unknown (Barlow et al. 2010), this finding of the complementary localization of the above-mentioned molecular species in the nuclear sub-compartments may shed light on the spatial organization of relevant cellular processes.

The presented method for multiple immunolabeling significantly advances the possibilities of stereological analysis of the mutual distribution of the molecules of interest in biological objects. Statistical evaluation of clustering and co-localization patterns and mapping of antigen distribution as areas with statistically significant labeling density provides valuable information about spatial interactions between biomolecules in situ (Mayhew and Lucocq 2011; Philimonenko et al. 2000; Schofer et al. 2004). While double-labeling enables analysis of one type of spatial interactions between different molecules and two types of interactions between the molecules of the same type, with the five types of labeling the possible number of assessed interactions increases up to ten and five, respectively, bringing more detailed understanding of the microarchitecture of functional domains in the cell. These interactions can be then quantitatively described using spatial statistics.

## Conclusion

Using newly synthesized nanoparticles conjugated to secondary antibodies, we demonstrated for the first time a simultaneous labeling of five biomolecules, making it possible to co-localize them in ‘one-go’ and thus more reliably predict their interplay in a cell or a tissue. The procedural advantage is that no complex equipment is required except a standard TEM, and only standard methodology is employed for specimen preparation. As current molecular research provides increasingly complex information about the interactions of various players in biological processes, new tools are expected to analyze the situation in situ, and we are presenting such an ultrastructural tool.
